# The Prognostic Significance of Immune-Related Metabolic Enzyme MTHFD2 in Head and Neck Squamous Cell Carcinoma

**DOI:** 10.3390/diagnostics10090689

**Published:** 2020-09-11

**Authors:** Li Cui, Huan Chen, Xinyuan Zhao

**Affiliations:** 1 Department of Oral and Maxillofacial Surgery, Stomatological Hospital, Southern Medical University, Guangzhou 510280, China; 2 Division of Oral Biology and Medicine, School of Dentistry, UCLA, Los Angeles, CA 90095, USA; 3 Department of Endodontics, Stomatological Hospital, Southern Medical University, Guangzhou 510280, China; huanchensmu@163.com

**Keywords:** immune-related metabolic enzymes, MTHFD2, head and neck squamous cell carcinoma, prognosis

## Abstract

Metabolic dysregulation has emerged as a crucial determinant of the clinical responses to immunotherapy. The aim of this study was to determine the clinical significance of the candidate immune-related metabolic enzymes (IRMEs) methylenetetrahydrofolate dehydrogenase (NADP+ dependent) 2 (MTHFD2) in head and neck squamous cell carcinoma (HNSCC). The gene expression profile of HNSCC cohort and the corresponding clinical information were downloaded from The Cancer Genome Atlas (TCGA). The differentially expressed IRMEs were identified, and then, the prognosis-associated IRMEs were revealed by univariate cox regression analysis. The prognostic significance of MTHFD2 in HNSCC as well as the association between MTHFD2 and immune cell infiltration were further analyzed. A total of 121 significantly altered IRMEs were identified between HNSCC and normal tissues, and 21 IRMEs were found to be strongly associated with overall survival of HNSCC. Upregulation of MTHFD2 was positively correlated with adverse clinicopathological factors in TCGA HNSCC cohort, which was further validated with our own cohort using immunohistochemical analysis. Interestingly, bioinformatic analysis further revealed that increased MTHFD2 expression was negatively associated with NK cells activation, while positively correlated with mast cells activation. In conclusion, MTHFD2 overexpression is closely correlated with unfavorable prognosis of HNSCC, and it might play an important role in modulating the tumor immune microenvironment.

## 1. Introduction

Head and neck squamous cell carcinoma (HNSCC) represents the sixth most common malignancy around the world [1,2,3]. It arises in the oral cavity, oropharynx, hypopharynx, and larynx [4]. Surgical resection, radiotherapy, chemotherapy, and targeted therapy remain the major treatment modalities for HNSCC. Accurate prediction of HNSCC prognosis is of great importance for successful clinical management and individualized medicine. The Tumor, Node, Metastasis (TNM) staging system for HNSCC is still the most widely used prognostic indicator in clinical practice. However, the HNSCC cases with the same TNM stage might have significantly different clinical outcomes. Therefore, identification of novel and robust prognostic biomarkers is critical for improving the prognosis of HNSCC. Recently, immunotherapy showed encouraging results for treating recurrent or metastasis HNSCC [5,6,7], indicating the modulating tumor immune microenvironment might contribute to improving the clinical outcome of HNSCC. Therefore, it is of great importance to identify the immune-related molecular biomarkers for HNSCC.

Understanding metabolic dysregulation in the tumor microenvironment is crucial for treating human cancer [8]. Many crucial metabolic pathways are dysregulated in cancer cells; thus, targeting the metabolism might be a good therapeutic strategy [9]. In addition, cancer cells are able to suppress anti-tumor immunity by modulating the metabolites in the tumor microenvironment or inhibit the metabolic fitness of tumor infiltrating immune cells. Therefore, metabolic interventions show great promise for enhancing the therapeutic effectiveness of immunotherapies [10]. Metabolic enzymes are important regulators that directly control the metabolic balance in the tumor microenvironment. Elucidating the role of immune-related metabolic enzymes (IRMEs) in HNSCC progression might provide important guidance for improving the therapeutic outcome. The Cancer Genome Atlas (TCGA) is a landmark cancer genomics program, which provides immeasurable and multi-dimensional data for 33 different types of cancer [11].

In this study, we first screened the significantly differentially expressed metabolic enzymes in TCGA HNSCC cohort. Then the IRMEs and the prognosis associated IRMEs were identified. An IRME-based prognostic signature was constructed to predict the prognosis of HNSCC. The clinical significance of methylenetetrahydrofolate dehydrogenase (NADP + dependent) 2 (MTHFD2) in HNSCC was further explored.

## 2. Materials and Methods

### 2.1. Data Source

The RNA-seq data of TCGA HNSCC cohort which included 502 HNSCC cases and 44 normal controls was downloaded from The National Cancer Institute Genomic Data Commons (NCI-GDC) (https://gdc.cancer.gov/). The list of metabolic enzymes was available from Mammalian Metabolic Enzyme Database. The differentially expressed metabolic enzymes between tumor samples and control samples were screened by the limma package. *p* < 0.05 and absolute log_2_FC > 1 was set as the cut-off threshold. The GSE6631 was downloaded from the NCBI GEO database (https://www.ncbi.nlm.nih.gov/geo/).

### 2.2. Gene Ontology (GO) and Pathway Enrichment Analysis

GO and Kyoto Encyclopedia of Genes and Genomes (KEGG) pathway enrichment analyses of the significantly changed IRMEs were performed using “clusterProfiler” and “enrichplot” R packages.

### 2.3. Identification of Immune-Related Metabolic Enzymes

The genes related to immune system process M13664 and immune response M19817 were downloaded from the Molecular Signatures Database (http://www.broadinstitute.org-/gsea/msigdb/index.jsp). Then Pearson’s correlation analysis was used to analyze the correlation between significantly differentially expressed metabolic enzymes and immune-related genes to identify the IRMEs. The absolute correlation coefficients ≥0.4 and *p* < 0.001 were set as the cut-off threshold.

### 2.4. IRME-Based Prognostic Signature Generation and Prediction

Univariate cox proportional hazards regression analysis was used to identify the IRMEs that strongly associated with the overall survival (OS) of HNSCC. The LASSO regression analysis was used to select the most optimal OS-associated proteins into the multivariate cox proportional hazards regression model. Then the prognosis-related IRMEs and their coefficients were determined with the multivariate analysis. A risk score for each patient was calculated as the sum of each IRME’s score, which was obtained by multiplying the expression level of the IRME and its corresponding coefficient. The TCGA HNSCC cohort was divided into high and low-risk groups with the median value of the risk scores. The differences in OS were compared between high and low-risk groups with the Kaplan–Meier method and log-rank test. Receiver operating characteristic (ROC) curve was constructed to evaluate the prediction accuracy of the IRME-based prognostic model.

### 2.5. Evaluation of Immune Cell Infiltration

The CIBERSORT algorithm was used to evaluate the immune cell infiltration in HNSCC samples. Immune cell fractions of each individual tumor sample were demonstrated in Supplementary Table S1. The median expression of MTHFD2 was used to divide the HNSCC cohort into high and low MTHFD2 expression groups. The differences in immune cell infiltration between high and low MTHFD2 expression groups were depicted with a violin plot. Pearson’s correlation analysis was used to analyze the association between MTHFD2 level and immune cell infiltration.

### 2.6. Tissue Samples and IHC Analysis

This study was approved by the Ethics Committee of the Stomatological Hospital, Southern Medical University. Written informed consent was obtained from all patients. A total of 87 HNSCC formalin-fixed paraffin-embedded (FFPE) specimens were included in this study. The clinicopathological information of the study cohort was summarized in Supplementary Table S2. For IHC analysis, FFPE blocks were deparaffinized by sequential washing with xylene, 100% ethanol, 95% ethanol, 80% ethanol, and PBS. Followed by quenching with 0.3% H_2_O_2_ in methanol for 5 min, the slides were incubated with 5% BSA for 30 min. Then the sections were probed with MTHFD2 primary antibody (1:150, ProteinTech, Chicago, IL, USA) overnight at the cold room. After washing with PBS, the slides were incubated with horseradish peroxidase linked secondary antibody for 2 h at room temperature. For the quantitative analysis, the staining score of MTHFD2 was calculated by multiplying the staining intensity (on a scale of 0–3: negative = 0, weak = 1, moderate = 2, and strong = 3) and the percentage of cells stained (on a scale of 0–4: 0 = 0%, 1 = 1–25%, 2 = 26–50%, 3 = 51–75%, and 4 = 76–100%).

### 2.7. Statistical Analysis

The data were subjected to normal distribution, expressed as the mean ± standard deviation and analyzed by the independent samples *t*-test. The Kaplan–Meier method and log-rank test were used for elucidating the association between MTHFD2 staining score and OS of HNSCC. Univariate and multivariate analyses were performed to identify the independent prognostic factors for HNSCC. Data analysis was performed with the GraphPad Prism 8.0 (GraphPad, San Diego, CA, USA). *p* value less than 0.05 was considered statistically significant.

## 3. Results

### 3.1. The Significantly Differentially Expressed Metabolic Enzymes in TCGA HNSCC Cohort

**Figure 1:**
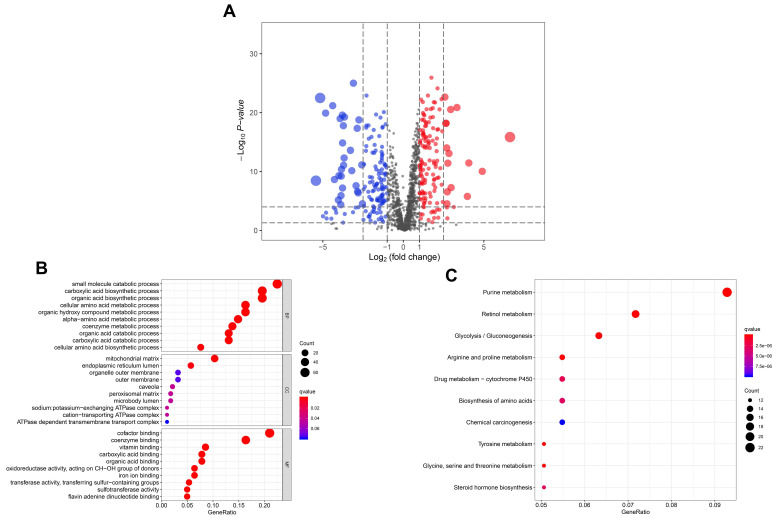
The significantly altered metabolic enzymes in The Cancer Genome Atlas (TCGA) head and neck squamous cell carcinoma (HNSCC) cohort. (**A**) Volcano plot of metabolic enzymes between HNSCC tissues and normal tissues. *Y*-axis indicates the *p* values (log_10_-scaled), whereas the *X*-axis indicates the fold change (log_2-_scaled). Each symbol represents a different metabolic enzyme. The red and blue color indicates the significantly upregulated and downregulated metabolic enzymes, respectively. (**B**,**C**) Gene Ontology (GO) and Kyoto Encyclopedia of Genes and Genomes (KEGG) analysis of the significantly altered metabolic enzymes.

A volcano plot was used to visualize the distribution of metabolic enzymes between tumor and control samples. Red or blue dots indicated the significantly upregulated or downregulated metabolic enzymes, respectively (Figure 1A). The detailed information of the significantly altered metabolic enzymes was listed in Supplementary Table S3. GO analysis of the differentially expressed metabolic enzymes revealed the top ten biological processes, cellular components, and molecular functions, which were shown in Figure 1B. Similarly, the most enriched signaling pathways were summarized in Figure 1C.

### 3.2. IRME-Based Prognostic Signature Generation and Prediction

**Figure 2:**
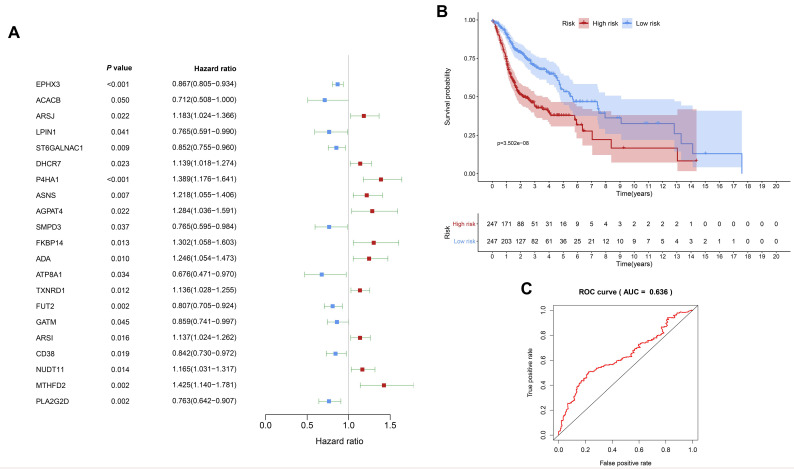
Immune-related metabolic enzyme (IRME)-based prognostic signature generation and prediction. (**A**) Univariate analysis revealed the OS-associated IRMEs. (**B**) An IRME-based prognostic was constructed. (**C**) The receiver operating characteristic (ROC) curve was used to evaluate the predictive accuracy of the IRME-based prognostic signature.

The IRMEs in HNSCC were identified and summarized in Supplementary Tables S4 and S5. Univariate Cox regression analysis was performed to identify the IRME that significantly associated with OS in TCGA HNSCC cohort. As shown Figure 2A, the hazard ratio of ATP8A1, ACACB, A2G2D, SMPD3, LPIN1, FUT2, CD38, ST6GALNAC1, GATM, and EPHX3 was less than 1, indicating they were protective genes for HNSCC. The hazard ratio of TXNRD1, ARSI, DHCR7, NUDT11, ARSJ, ASNS, ADA, AGPAT4, FKBP14, P4HA1, and MTHFD2 was larger than 1, suggesting that they were risky genes for HNSCC.

Based on the LASSO algorithm, EPHX3, LPIN1, P4HA1, ADA, CD38, NUDT11, MTHFD2, and PLA2G2D were selected to construct the prognostic signature. The risk score for each patient was calculated with the following formula: risk score = EPHX3 × (−0.099) + LPIN1 × (−0.271) + P4HA1 × (0.141) + ADA × (0.150) + CD38 × (−0.186) + NUDT11 × (0.165) + MTHFD2 × (0.378) + PLA2G2D × (−0.159). The survival analysis demonstrated that the HNSCC patients in the high-risk group suffered a significantly shorter OS than those in the low-risk group (*p* = 3.502 × 10^−8^ (Figure 2B). For the predicting accuracy, the area under the ROC curve (AUC) value of the IRME-based prognostic model was 0.636 (Figure 2C).

### 3.3. Upregulation of MTHFD2 Was Associated with Poor Clinical Outcome of HNSCC in the Public Datasets

**Figure 3:**
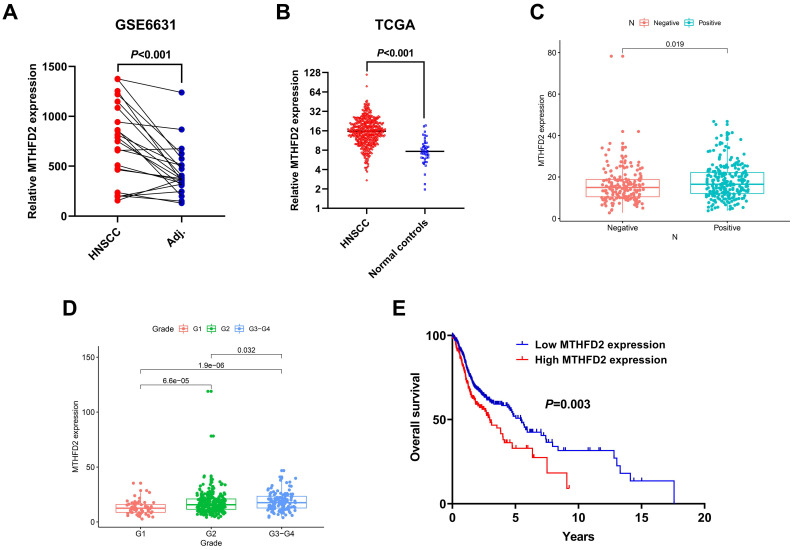
Upregulation of methylenetetrahydrofolate dehydrogenase (NADP+ dependent) 2 (MTHFD2) was associated with poor clinical outcome of HNSCC in the public datasets. (**A**) MTHFD2 was overexpressed in HNSCC tumor specimens compared to the paired adjacent normal tissues. (**B**) MTHFD2 was upregulated in HNSCC tissues compared to the normal controls. (**C**) High MTHFD2 expression was positively associated with lymph node metastasis. (**D**) High MTHFD2 expression was positively correlated with tumor grade. (**E**) Patients in the high MTHFD2 group had worse OS than those in the low MTHFD2 group.

The expression level of MTHFD2 was significantly overexpressed in tumor tissues compared to the normal controls in both GSE6631 and TCGA HNSCC cohort (Figure 3A,B). In addition, for the TCGA HNSCC cohort, upregulation of MTHFD2 was positively correlated with positive lymph node metastasis and advanced tumor grade (Figure 3C,D). The HNSCC patients with higher MTHFD2 expression had a worse OS than those with lower MTHFD2 expression (*p* = 0.003) (Figure 3E).

### 3.4. The Association between MTHFD2 and Immune Cell Infiltration in HNSCC

**Figure 4:**
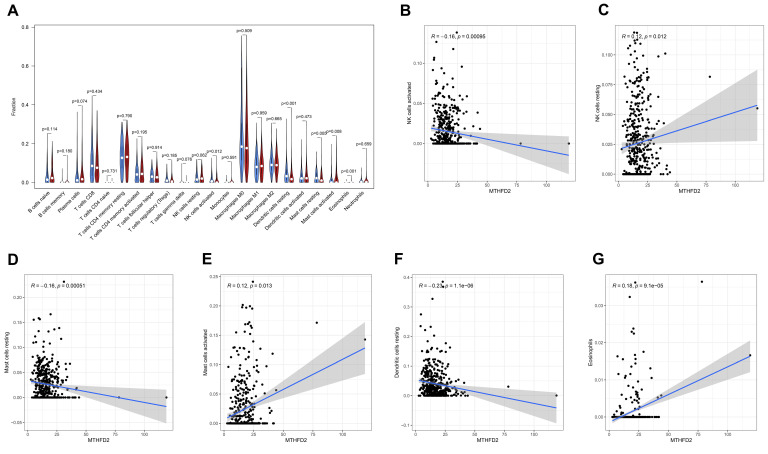
The association between MTHFD2 and immune cell infiltration in HNSCC. (**A**) higher fractions of mast cells activated and eosinophils were observed in the high MTHFD2 expression group, while higher fractions of NK cells activated, dendritic cells resting, and mast cells resting were found in the low MTHFD2 expression group. (**B**–**G**) MTHFD2 was positively correlated with NK cells resting, mast cells activated, and eosinophils, while negatively associated with NK cells activated, mast cells resting, and dendritic cells resting.

As shown in Figure 4A, higher fractions of mast cells activated and eosinophils were observed in the high MTHFD2 expression group, while higher fractions of NK cells activated, dendritic cells resting, and mast cells resting were found in the low MTHFD2 expression group. The Pearson correlation analysis revealed that MTHFD2 was positively correlated with NK cells resting, mast cells activated, and eosinophils, while negatively associated with NK cells activated, mast cells resting, and dendritic cells resting (Figure 4B,G).

### 3.5. The Prognostic Value of MTHFD2 in HNSCC Was Validated in an Independent Cohort

**Figure 5:**
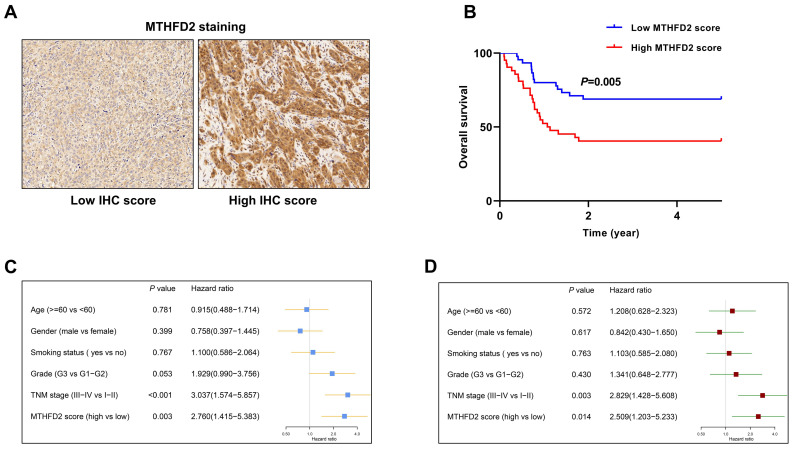
The prognostic value of MTHFD2 in HNSCC was validated in an independent cohort. (**A**) The representative samples in the high and low IHC score groups. (**B**) The patients in the high MTHFD2 score group had shorter OS than those in the low MTHFD2 score group. (**C**) Univariate analysis showed that MTHFD2 score was significantly associated with OS of HNSCC. (**D**) Multivariate analysis revealed that MTHFD2 score was an independent risk factor for HNSCC.

The staining score of MTHFD2 is calculated for each tumor sample. The median value of IHC score is used as the cut-off point to split the study cohort into high and low MTHFD2 groups. The representative samples in the high and low IHC score groups were demonstrated in Figure 5A. MTHFD2 score was higher in patients at the advanced stage or with advanced tumor grade (Table 1). The survival analysis showed that the patients in the high MTHFD2 score group had significantly shorter OS than those in the low MTHFD2 score group (*p* = 0.005) (Figure 5B). The univariate analysis showed that TNM stage and MTHFD2 score was strongly associated with the OS of HNSCC (Figure 5C). The multivariate analysis revealed that TNM stage and MTHFD2 score were independent risk factors for HNSCC (Figure 5D).

## 4. Discussion

Bioinformatic analysis of the significantly altered metabolic enzymes revealed many important metabolic pathways were deregulated in HNSCC. For instance, purine metabolism and biosynthesis of amino acids provide the essential nutrients for maintaining the oncogenic activities of cancer cells [12,13]. It is widely observed that many cancer cells display enhanced glycolytic activity and suppressed mitochondrial metabolism [14], which is consistent with our findings that glycolysis/gluconeogenesis was the top enriched pathway in HNSCC.

A number of IRMEs were identified in HNSCC, suggesting that aberrant expression of IRMEs might modulate the tumor immune microenvironment. A total of 21 IRMEs were significantly associated with OS of HNSCC. Among them, 10 were protective IRMEs, while the remaining 11 IRMEs were risky genes. High P4HA1 mRNA level was reported to be significantly associated with unfavorable OS and locoregional recurrence of oral squamous cell carcinoma [15], indicating that P4HA1 might act as a potent tumor promoter. In addition, we have constructed an IRME-based prognostic signature for predicting the clinical outcome of HNSCC, indicating that abnormal expression pattern of IRMEs is closely correlated with the survival of HNSCC. However, further studies are needed to perform to validate the robustness of this IRME-based prognostic model.

MTHFD2, which is in the prognostic signature, was chosen for further exploration. The expression level of MTHFD2 was elevated in HNSCC, and upregulation of MTHFD2 was closely correlated with poor prognosis of HNSCC, indicating that MTHFD2 might play a tumor promoting role in the progression of HNSCC. To the best of our knowledge, this is the first report to elucidate the clinical significance of MTHFD2 in HNSCC. Consistent with our results, Pikman et al. reported that MTHFD2 was overexpressed in acute myeloid leukemia (AML). In addition, downregulation of MTHFD2 reduced growth and colony formation and induced differentiation in AML cells [16]. Interestingly, knockdown of MTHFD2 in breast cancer cells significantly affected many important metabolic pathways, suggesting that MTHFD2 might be a central metabolic enzyme in cancer cells [17]. Interestingly, MTHFD2 is important for maintaining global N6-methyladenosine (m^6^A) methylation levels in renal cell carcinoma (RCC). MTHFD2 promoted the translation of HIF-2α by m^6^A methylation of HIF-2α mRNA, which results in promoting metabolic reprograming and tumor growth [18]. In addition to its known function in mitochondrial folate metabolism, Nina reported that MTHFD2 was able to enter the nucleus and co-localize with DNA replication sites [19], which further supports its important regulatory roles in tumorigenesis. Costas also demonstrated that MTHFD2 interacted with many nuclear proteins associated with RNA metabolism and translation [20]. Further studies are warranted to elucidate the molecular mechanisms for the tumor promoting role of MTHFD2 in HNSCC.

Interestingly, we found that MTHFD2 upregulation was positively correlated with NK cells resting and mast cells activated, while negatively associated with NK cells activated and mast cells resting. These results indicate that MTHFD2 might play an important role in modulating the tumor immune microenvironment. NK cells are innate immune cells with potent cytolytic activity against tumors [21,22]. Upregulation of MTHFD2 might result in increased NK cells resting and decreased NK cells activated, suggesting that MTHFD2 might exert its tumor promoting by affecting the activities of NK cells. Mast cells play multifaceted roles in regulating the tumor microenvironment [23]. They can either suppress or promote anti-tumor immunity [24]. Considering the potential tumor promoting role of MTHFD2 in HNSCC, activation of mast cells might suppress anti-tumor immunity in HNSCC. Immunotherapy has shown great promise for the treatment of HNSCC. Both nivolumab and pembrolizumab are monoclonal antibodies that block PD-1 and have been approved by FDA for treating relapsed or metastatic HNSCC when the patients are resistant to cisplatin [6]. However, many factors such as tissue PD-L1 expression, tumor mutational burden, and the viral etiology might greatly affect the efficacy of immunotherapy [25]. As our results show that MTHFD2 is an IRME, it is very interesting to investigate whether targeting MTHFD2 could enhance the effectiveness of immunotherapy.

Age, gender, smoking status, tumor grade and TNM stage are potential prognostic indicators for HNSCC. As shown in Figure 5D, the risk ratio of MTHFD2 was 2.509, which was larger than that of age, gender, smoking status, and tumor grade, but smaller than that of TNM stage. The heterogeneous attribute of HNSCC complicates its accurate prognostication. Therefore, combining the TNM stage and other prognostic indicators such as MTHFD2 score might contribute to improve the prognosis prediction of HNSCC.

In conclusion, we have identified the IRMEs and OS-associated IRMEs in HNSCC. In addition, an IRME-based prognostic signature is built up. Moreover, MTHFD2 is overexpressed in HNSCC, and upregulation of MTHFD2 is correlated with worse clinicopathological parameters and clinical outcome of HNSCC. MTHFD2 overexpression might be also associated with the abnormal immune microenvironment. Collectively, MTHFD2 might serve as a promising therapeutic target for HNSCC.

## Figures and Tables

**Table 1:** The correlation between MTHFD2 staining score and clinicopathological parameters of HNSCC.

Variables	Staining Score	Min/Max	*p*
**Age**			0.801
≥60	7.222 ± 3.819	0/12	
<60	7.429 ± 3.781	2/12	
**Gender**			0.460
Male	7.508 ± 3.856	0/12	
Female	6.833 ± 3.608	2/12	
**Smoking status**			0.199
No	7.944 ± 3.561	2/12	
Yes	6.882 ± 3.902	0/12	
**Grade**			0.007
G1–G2	6.576 ± 3.979	0/12	
G3	8.893 ± 2.780	3/12	
**TNM stage**			0.002
I–II	6.174 ± 3.641	0/12	
III–IV	8.610 ± 3.549	2/12	

## Supplementary Materials

The following are available online at https://www.mdpi.com/2075-4418/10/9/689/s1. Table S1: The relative immune cell infiltration in HNSCC tumor samples; Table S2: The clinical information of the study cohort; Table S3: The significantly changed metabolic enzymes between HNSCC tumor tissues and the control tissues; Table S4: Identification of the immune-related metabolic enzymes in HNSCC; Table S5: The significantly differentially expressed immune related metabolic enzymes in HNSCC.
